# Future Medical Doctors Are Not Learning About Overweight and Obesity in Children: Curriculum Analysis at Five Australian Medical Schools

**DOI:** 10.1007/s40670-025-02527-0

**Published:** 2025-10-23

**Authors:** Emma Schwartzkoff, Terri Pikora, Hannah Garven, Michelle Gooey, Conor Gilligan, Jennifer Lindley, Nicola Kerr, Lillian Smyth, Gordana Popovic, Gina Arena, Linda Ferrington

**Affiliations:** 1 Mid North Coast Local Health District, Health Promotion, Port Macquarie Community Health, Port Macquarie, NSW 2444 Australia; 2 School of Health Sciences (Nutrition and Dietetics), University of Newcastle, University Drive, Callaghan, NSW 2308 Australia; 3 Rural Clinical School of Western Australia, The University of Western Australia, 35 Stirling Terrace, Albany, WA 6330 Australia; 4 School of Clinical Medicine, University of New South Wales, Rural Clinical Campus Port Macquarie, 20 Highfields Circuit, Port Macquarie, NSW 2444 Australia; 5 Health and Social Care Unit, School of Public Health and Preventive Medicine, Monash University, 553 St Kilda Road, Melbourne, VIC 3004 Australia; 6 Faculty of Health Sciences and Medicine, Bond University, 14 University Drive (Off Cottesloe Drive) Robina , Gold Coast, QLD 4226 Australia; 7 School of Medicine and Psychology, Australian National University, Florey Building 54 Mills Road, Canberra, ACT 2601 Australia; 8 Stats Central, Mark Wainwright Analytical Centre, University of New South Wales, Kensington, Australia; 9 Medical School, The University of Western Australia (UWA), Perth, Australia

**Keywords:** Childhood obesity, Overweight, Obesity, Medical school curricula

## Abstract

This short communication evaluates the inclusion of content about overweight and obesity in children within the curricula of five Australian medical schools. Despite the rising prevalence of overweight and obesity in children and its significant health impacts, the analysis reveals substantial gaps in the coverage of obesity-related topics, particularly those focused on children. The findings highlight the need for a more consistent and structured approach to obesity education in medical training. Enhancing curricula to include comprehensive obesity education, especially related to childhood, is crucial for preparing future doctors to address this growing public health issue effectively and sensitively.

**Supplementary Information:**

The online version contains supplementary material available at 10.1007/s40670-025-02527-0.

## Background

Comprehensive medical curricula and well-defined learning outcomes ensure focussed instruction and assessment [1] so future doctors are well prepared to meet current healthcare challenges. To remain relevant amid evolving population needs and advancing evidence, medical curricula should be regularly evaluated.

Overweight and obesity represent an evolving health challenge shaped by the environmental, social and economic realities of modern life which collectively contribute to an obesogenic environment [2]. Globally, overweight and obesity rates in children and adolescents quadrupled over the past 30 years. In Australia, more than one in four children live with either overweight or obesity [3]. Clinical obesity is a condition or illness resulting from excess adiposity on the function of organs and tissues [4]. Clinical obesity in childhood and adolescence has been associated with immediate and long term health outcomes [2]. Additionally, weight stigma further impacts physical and mental health [5], compounding the overall health burden. To reduce the burden of disease over a lifetime and improve health outcomes for future generations, early lifestyle intervention in childhood is essential, as is addressing and reducing weight stigma [6, 7].

Despite the importance of clinical obesity, previous research suggests that many healthcare professionals feel underprepared to address overweight and obesity, especially in children, often lacking the necessary skills and confidence [8]. This raises concerns about whether medical education provides sufficient training in this area. While this issue has been identified by several researchers [9–11], there remains limited research that systematically evaluates obesity related content, particularly content focussed on children and adolescents, in current medical school curricula.

The purpose of the study described in this short communication was to evaluate curricula of Australian medical schools for content related to overweight and obesity in children and adolescents. The evaluation was conducted against pre-defined learning outcomes developed through consultation with literature, field experts and people with lived experience of obesity (unpublished manuscript, under review). Identifying gaps in existing curricula can support more targeted curriculum planning and ultimately improve obesity education in medical training nationwide.

## Activity

**Fig. 1:**
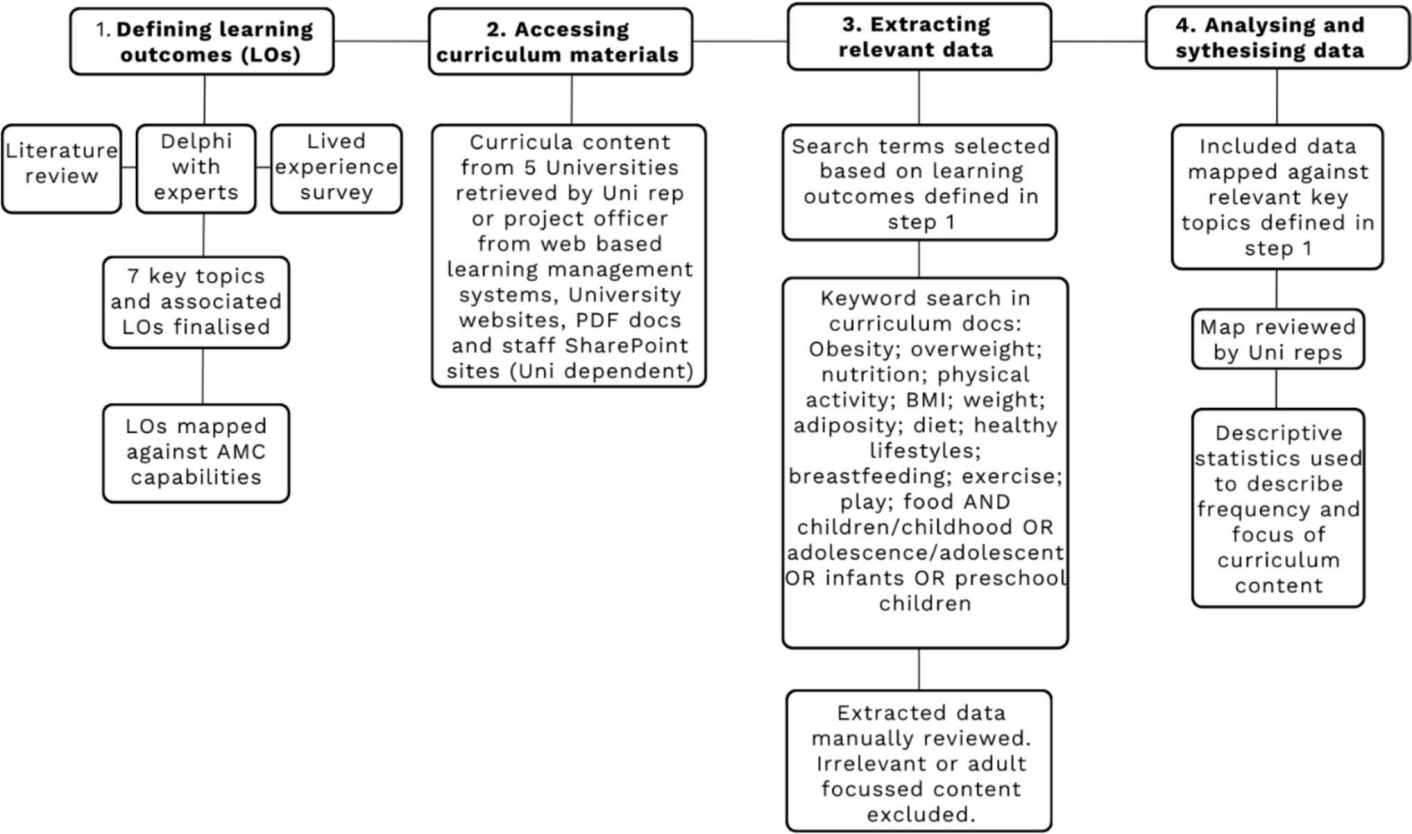
Flowchart of the curriculum content review process based on the document review method by Rohwer et al. (2017), as applied to overweight and obesity in children

This study used a retrospective content analysis to evaluate curricula from five Australian medical schools across four Australian states for content related to overweight and obesity in children. The systematic document review process was based on the process outlined by Rohwer et al. [12]. See Fig. 1 for the evaluation process.

As outlined in Fig. 1, the seven key topics forming the “ideal curriculum” (Table 1) were developed through a multi-stage process. A narrative review informed a draft set of learning outcomes, which were refined and prioritised through a modified Delphi process with clinicians, academics, public health professionals and policy makers. Input from people with lived experience of obesity further guided the wording and selection, resulting in the final seven learning outcomes. The full curriculum development process in described in detail elsewhere (unpublished manuscript, under review); here, it is presented only as background for the subsequent evaluation. For the curriculum evaluation, we used key word searches to identify specific mentions of relevant terms including childhood obesity; obesity; overweight; nutrition; physical activity; BMI; weight; adiposity; diet; healthy lifestyles; breastfeeding; exercise; play; food AND children/childhood OR adolescence/adolescent OR infants OR preschool children or the terms used separately in association with each other in the context of a curricula item (timetabled session or resource). The information, including a description of the content taught and the teaching/learning modality retrieved for each university was collated into a standardised data collection form in Excel and mapped against the ideal learning outcomes by one researcher (TP). The completed evaluation tool was cross checked by representatives from each University. Descriptive statistics were then used to describe the topics covered and the frequency of coverage.

**Table 1:** Frequency of key topics identified by keyword search and the teaching modality used in the curriculum documents of Australian medical schools

Topic*	University				
	#1	#2	#3	#4	#5
Impact of overweight and obesity in children including health outcomes and societal impact	-	-	1^−^	-	1^#^
The concept of “obesogenic environments” and strategies to modify them	1^%^	1^%^	-	1^$^	1^#^
Risk factors contributing to overweight and obesity in children and adolescents	1^%^	1^%^	3^^&−^	1^$^	1^#^
Screening practices to identify children and adolescents with or at risk of developing overweight or obesity	-	3^&#@^	3^^+−^	-	-
Strategies for prevention and management of overweight and obesity in children and adolescents	1^%^	1^%^	3^^&−^	-	1^#^
Sensitive, non-judgemental and effective communication techniques	1^%^	1^@^	3^^+&^	-	-
Factors contributing to and implications of weight bias and weight stigma	-	-	1^^^	-	-
**Total**	**4**	**7**	**14**	**2**	**4**

*Topics are aligned with an “ideal” curriculum framework (available as supplementary material 1), and the table indicates how often each topic is referenced by each university across all years of their program

^#^Self-directed; ^^^Didactic; ^+^Clinical based; ^%^Case studies; ^$^Discussion; ^&^Tutorial; @Practice-based assessment; ^−^Unknown

### Compliance with Ethical Standards

This research project was carried out in accordance with the Declaration of Helsinki. Ethics approval was provided by the UNSW Research Ethics Office (reference HC230110) and ratified by each of the universities involved.

The authors have no competing interests to declare that are relevant to the content of this article.

## Results

Across the entire medicine curriculum, no university covered all seven recommended topics from the ideal curriculum. There was considerable heterogeneity in childhood specific obesity content between universities. University 3 demonstrated the most comprehensive coverage, mentioning overweight and obesity in children 14 times and addressing six of the seven recommended topics (see Table 1). In contrast, University 4 mentioned overweight and obesity just twice across two of the seven topics. Obesity in childhood was mentioned seven times in the curriculum from university 2, across five of the seven topics, while universities 1 and 5 each recorded four instances across four topics.

## Discussion

This short communication identified a lack of coverage of key obesity-related topics specific to children in a comprehensive review of five Australian medical degree programmes. All five Australian medical schools in this study addressed overweight and obesity to some extent in their curricula, but there was considerable variability in coverage (Table 1).

These findings about content variability highlight the need for more consistent inclusion of content relating to overweight and obesity in childhood in medical curricula. The Australian Medical Council develops accreditation standards for medical schools [13]. The standards define the qualities expected of graduating doctors but do not dictate curricula content. This is determined by individual universities and can be contentious due to the input from a large number of stakeholders with different priorities [14]. This likely contributes to the content variability between institutions observed in this short communication. This issue is not easily resolved, with university medical schools facing curriculum overload [15] driven by continual influx of content deemed ‘essential’ [16]. Developing clear Australian national guidelines could provide clarity as to which critical obesity topics are consistently addressed across medical schools given the multiple curriculum priorities.

The lack of obesity-related topics focussed on children is particularly concerning given the critical role that early intervention and prevention play in addressing the obesity epidemic [6] as well as the interest that Australian health providers have shown in delivering preventive care relating to overweight and obesity in children [17]. Ensuring that curricula include child-specific content on obesity may help to foster a paradigm shift away from reactive healthcare towards proactive prevention strategies.

This study highlights a critical gap in medical education regarding weight bias and stigma which was the least frequently mentioned topic throughout the curricula. This is concerning given that previous research has shown that both medical students and doctors exhibit high levels of weight bias [18, 19], which can negatively affect the quality of care patients receive [18]. In line with these findings, previous research has recommended incorporating patient counselling skills and initiatives to address physicians’ attitudes toward patients with obesity into medical curricula [10].

## Limitations and Future Directions

The scope of the current study was limited to content analysis, and it is important to note that variations in universities' approaches to curriculum mapping due to different content storage systems may have resulted in missed items, which might contribute to the differences observed in obesity-related content. However future research should explore strategies for integrating obesity related content into existing curricula. This includes consideration of timing, mode of delivery and alignment with current topics to ensure such integration enhances learning rather than adds to the burden of an already crowded curriculum.

## Conclusion

This study identified major gaps in the curricula of five Australian medical schools, which may indicate that dedicated education about overweight and obesity in children is deficient across all Australian medical school curricula. These findings underscore the urgent need for medical schools to review and update their curricula to include a stronger focus on obesity, particularly in children. Medical schools should use this work to pursue opportunities to develop their curricula and ensure they produce graduates with the skills and knowledge to address this critical issue of healthcare.

## Supplementary Information

Below is the link to the electronic supplementary material.

ESM 1 (DOCX 24.0 KB)
