# Supplementary material for: Future Medical Doctors Are Not Learning About Overweight and Obesity in Children: Curriculum Analysis at Five Australian Medical Schools
Source: Med Sci Educ. 2025 Oct 23;35(6):2793–7. doi: 10.1007/s40670-025-02527-0 (PMC12961014; doi:10.1007/s40670-025-02527-0)
Supplement: Supplementary file 1 — (DOCX 24.0 KB) [file 40670_2025_2527_MOESM1_ESM.docx]

Supplementary Material 1

Table 2 Final Learning Outcomes

| *Demonstrate Knowledge and Understanding of* | *Be Able To* |
| --- | --- |
| 1.1 the health outcomes related to overweight and obesity in children and adolescents. | 1.3 apply knowledge of obesity-related health outcomes to clinical scenarios. |
| 1.2 the societal impact of overweight and obesity in children and adolescents. | 1.4 sensitively initiate discussions with children, adolescents and/or caregivers about obesity-related health outcomes. |
| 2.1 the concept of “obesogenic environments”. | 2.2 identify the impact of obesogenic environments on children, adolescents and families in real life scenarios. |
|  | 2.3 propose interventions and strategies aimed at modifying obesogenic environments to promote healthier behaviours and reduce the prevalence of overweight and obesity in children and adolescents. |
| 3.1 risk factors contributing to overweight and obesity in children and adolescents. | 3.2 conduct a thorough medical and social history to identify factors that may increase a child or adolescent’s risk of developing overweight or obesity. |
| 4.1 routine screening practices to identify children and adolescents with or at risk of developing overweight or obesity. | 4.2 conduct accurate growth assessments and interpret growth charts to screen for overweight and obesity in children and adolescents. |
|  | 4.3 sensitively communicate findings from growth assessments to children, adolescents and their carers. |
| 5.1 evidence based strategies for the prevention and management of overweight and obesity in children and adolescents, including the importance of a multidisciplinary approach. | 5.2 develop family focussed management plans for families with children or adolescents at risk of or with overweight or obesity. |
| 6.1 factors that may impact a strong clinical alliance with children or adolescents who have overweight or obesity and their carers. | 6.2 use sensitive, non-judgemental language to communicate respectfully and compassionately with children and adolescents who have overweight or obesity and their carers. |
|  | 6.3 Use motivational interviewing techniques to support behaviour change among children and adolescents and their carers. |
| 7.1 the psychological, social, and cultural factors contributing to weight stigma and weight bias. | 7.3 critically reflect on personal beliefs, attitudes, and experiences related to weight and body image. |
| 7.2 the implications of weight stigma, weight bias and individual blame on the health and care outcomes for children and adolescents with overweight or obesity and their carers. |  |
